# Preferential Accumulation of Phospholipid-PEG and Cholesterol-PEG Decorated Gold Nanorods into Human Skin Layers and Their Photothermal-Based Antibacterial Activity

**DOI:** 10.1038/s41598-019-42047-7

**Published:** 2019-04-08

**Authors:** Nouf N. Mahmoud, Ala A. Alhusban, Jamila Isabilla Ali, Amal G. Al-Bakri, Rania Hamed, Enam A. Khalil

**Affiliations:** 1 Department of Pharmacy, Faculty of Pharmacy, Al-Zaytoonah University of Jordan, Amman, 11733 Jordan; 2 Department of Pharmaceutics & Pharmaceutical Technology, School of Pharmacy, The University of Jordan, Amman, 11942 Jordan

## Abstract

Herein, a library of gold nanorods (GNR) decorated with polyethylene glycol-thiol (PEG-SH) containing different functionalities were synthesized and characterized by optical absorption spectroscopy, zeta potential, dynamic light scattering (DLS), transmission electron microscope (TEM) and proton nuclear magnetic resonance (^1^H-NMR). The colloidal stability of GNR when exposed to skin, and their preferential accumulation into excised human skin layers were investigated. Confocal laser scanning microscopy, transmission electron microscope (TEM) and inductively coupled plasma-optical emission spectroscopy (ICP-OES) were utilized to track the penetration of GNR into different skin layers. The results demonstrated that cholesterol-PEG coated GNR were preferentially loaded up in the upper layers of skin (stratum corneum), while phospholipid-PEG coated counterparts were drastically deposited in skin dermis. Neutral methoxy-PEG-coated GNR were distributed in both SC and dermis skin layers, while charged GNR (anionic-carboxylic acid-PEG-GNR and cationic-amine-PEG-GNR) revealed a minimal accumulation into skin. DSPE-PEG-GNR and Chol-PEG-GNR demonstrated antibacterial activities against *Staphylococcus aureus* (*S aureus*) at MIC values of 0.011 nM and 0.75 nM, respectively. Photothermal treatment for *S. aureus* at sub-MIC concentrations resulted in a significant bactericidal effect when using Chol-PEG-GNR but not DSPE-PEG-GNR. Gold-based nanoscale systems have great value as a promising platform for skin diseases therapy.

## Introduction

Skin is considered as the ideal site for non-invasive skin therapeutic platforms^1,2^. Stratum corneum (SC) consists of proteins and lipid matrix and limits the penetration of the topically applied drugs^3–5^. Chemical and physical penetration enhancers are commonly used to modulate the skin morphology at the molecular level^6,7^. Furthermore, dermal and transdermal drug delivery nanocarriers, such as polymers, liposomes, lipids and inorganic nanoparticles, can retard drugs’ degradation, prolong their release, and enhance their solubility^8–11^.

Among nano-systems, gold nanoparticles (GNP) and in particular, gold nanorods (GNR) have important applications in nanomedicine such as imaging, sensing, bio-tracking and photothermal therapy owing to their optical properties^12–15^. GNP of different shapes, sizes and surface functionalities were employed to probe the skin-nano interface^16^. For example, Sonavane *et al*. have demonstrated that the small-sized GNP penetrated into skin dermis^17^, whereas Fernandes *et al*. revealed that GNP with rod-like shape have better penetration through skin compared to spherical counterparts^18^. Besides, negatively-charged skin components have repelled anionic nanoparticles, while higher affinity to SC has been reported using cationic nanoparticles^19^. Moreover, the surface modification of nanoparticles, such as proteins, lipids and oleic acids has increased the permeability and accumulation of nanoparticles into different skin compartments^18,20–23^.

Phospholipids, the main component of the cell membrane, have been used as a biocompatible surface coating agent for many nanoparticles such as graphene, silica, inorganic nanoparticles and quantum dots^24–27^. Lipid-coated nanomaterials have great potentials for biomedical applications. For example, clusters of phospholipid-coated GNP and lipid-coated GNP have been used to enhance the theranostic effects, cellular uptake, drug delivery and cancer therapy of GNP^28–32^.

Accumulation of nanomaterials at specific skin sites has a great impact on dermal drug delivery and tracking, in addition to their applications in diagnosis and skin diseases therapy^33^. In our earlier work, hydrophobic GNR coated with polystyrene-thiol were designed to be preferentially accumulated into human skin hair follicles, with minimal dermal distribution, as a promising platform for photothermal treatment of acne^34^. However, the interaction of phospholipid and cholesterol-modified-GNR with human skin and their accumulation potentials into skin layers have not been investigated fully in the literature.

The unique photothermal properties of GNR make them attractive anticancer and antibacterial candidates. GNR can convert the absorbed light into intense localized heat that could be effective for cancer and bacterial photothermal ablation^15^. Recently, the photo-thermolysis activity of GNR of hydrophilic or hydrophobic surface properties was evaluated against common skin bacteria such as *Propionibacterium acnes* and *Staphylococcus aureus* (*S. aureus*)^35^. In addition, photothermal ablation was reported for GNR conjugated to antibodies against *Pseudomonas aeruginosa*^36^.

Taking into consideration the unique advantages of GNR as a “nano-model”, their superiority in synthesis, colloidal stability, drug delivery and therapy, GNR decorated with PEG-thiol containing different functionalities were synthesized in this study, and their colloidal stability upon human skin contact was investigated. Employing confocal laser scanning microscopy, transmission electron microscopy (TEM) and inductively coupled plasma-optical emission spectroscopy (ICP-OES), the skin penetration and preferential accumulation of GNR decorated with phospholipid or cholesterol conjugated to PEG-thiol (SH) were probed and compared to that of GNR decorated with methoxy (m)-PEG or charged PEG moieties (carboxylic acid or amine groups). The effect of particle size and vehicle on the accumulation of GNR into skin layers was evaluated. Moreover, the antibacterial and photothermal-based antibacterial activities of DSPE-PEG-GNR and Chol-PEG-GNR were evaluated against *S. aureus*, a common skin pathogen.

## Results and Discussion

### Synthesis and characterization of GNR of different particle sizes and coated with PEG-SH-containing different functionalities

CTAB-assisted synthesis protocol was used to prepare GNR having aspect ratio (length/width; AR) of ~4 and ~2. This particular shape of GNP was selected owing to their uncommon optical characteristics, superior photothermal and antibacterial activities, and their potential accumulation into skin layers^13,15,18,35,37,38^.

**Figure 1:**
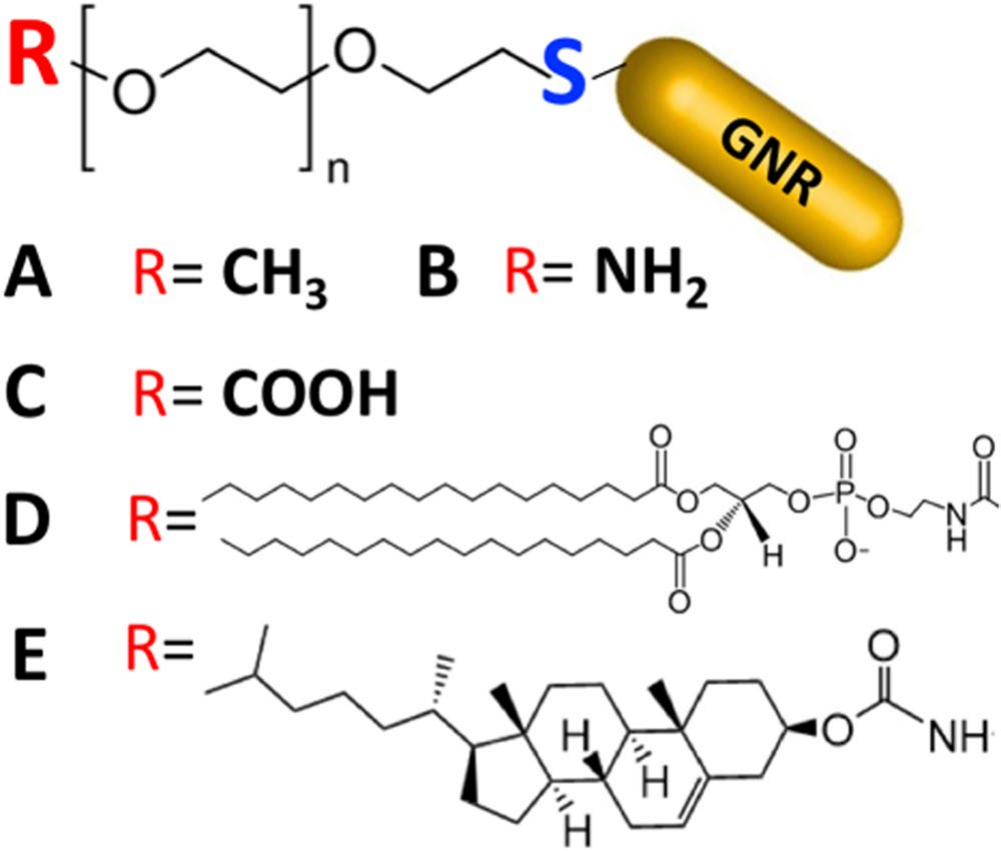
Illustration of different ligands containing PEG-SH used to functionalize the surface of GNR. (**A**) Methyl; (**B**) Amine; (**C**) Carboxylic acid; (**D**) Phospholipid (DSPE); and (**E**) Cholesterol.

**Figure 2:**
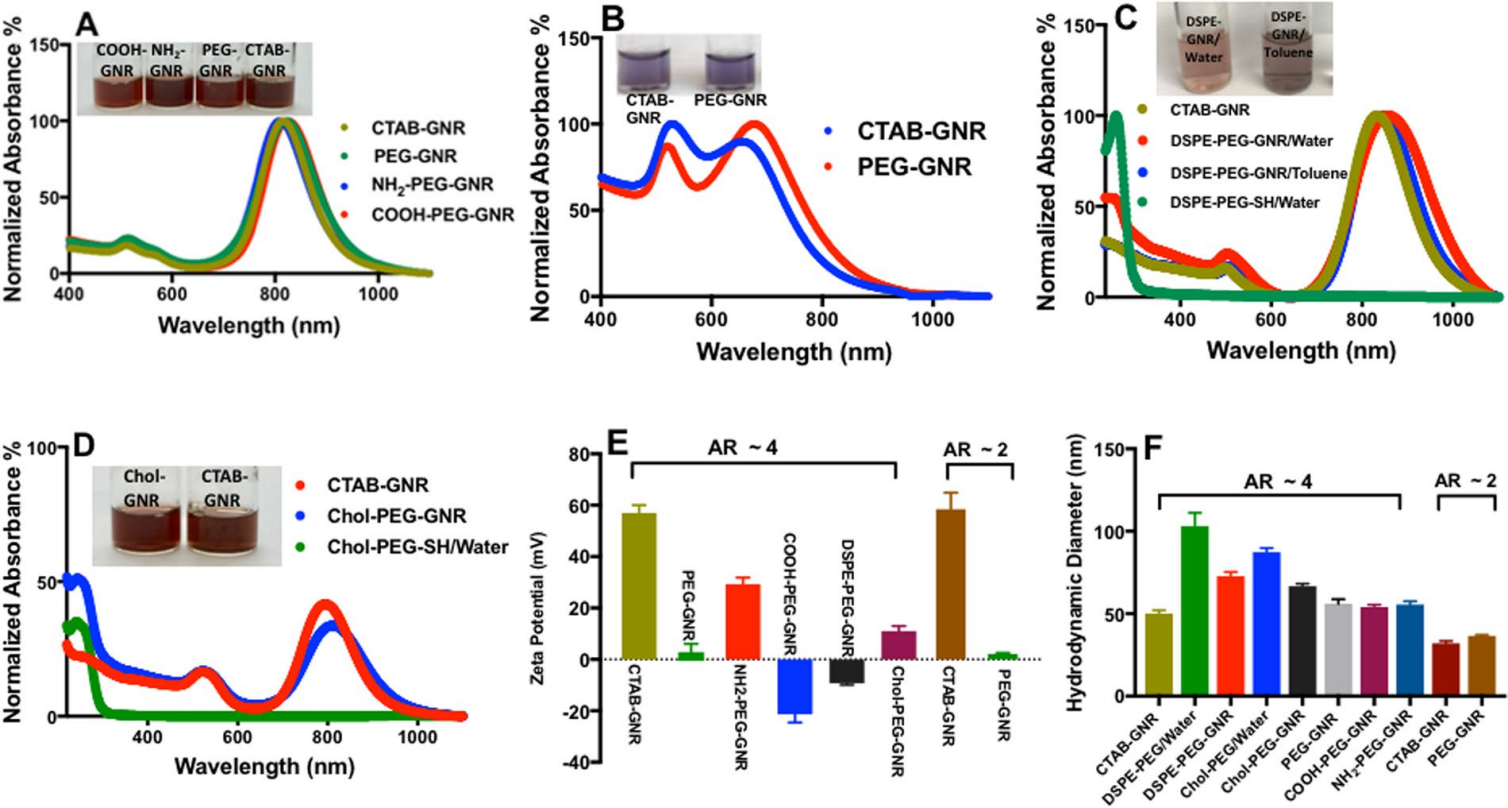
Characterization of GNR of different particle sizes and surface functionalities. (**A**) Optical absorption spectra and suspension photos of CTAB-GNR, m-PEG-GNR, NH_2_-PEG-GNR and COOH-PEG-GNR (AR ~ 4). (**B**) Optical absorption spectra and suspension photos of CTAB-GNR and PEG-GNR (AR ~ 2). (**C)** Optical absorption spectra and suspension photos of CTAB-GNR, DSPE-PEG-GNR/water, DSPE-PEG-GNR/toluene and DSPE-PEG-SH/water. (**D**) Optical absorption spectra and suspension photos of CTAB-GNR, Chol-PEG-GNR and Chol-PEG-SH/water. (**E**) Zeta potentials of GNR of different particle sizes and surface functionalities (**F**) Hydrodynamic diameter of GNR of different particle sizes and surface functionalities measured by DLS (nm). Data in E and F are presented as mean ± SD (n = 3). AR: aspect ratio.

Due to the cellular toxicity of CTAB molecules^39^, CTAB bilayers of GNR were replaced by ligand exchange using thiol-containing polyethylene glycol (PEG-SH). PEG-SH was utilized in this study as a binding ligand on GNR to facilitate the deposition of other functional moieties on the GNR surface *via* Au—S bonding (Fig. 1). PEG-SH is mostly used in ligand exchange reactions with GNR due to its high affinity to gold, well-known biocompatibility, and its key role in enhancing the GNR colloidal stability and disperse-ability in aqueous media^40–42^. Neutral m-PEG-coated GNR, anionic-COOH-PEG-coated GNR, cationic-NH_2_-PEG-coated GNR, phospholipid (DSPE)-PEG coated-GNR (suspended in water or toluene) and Chol-PEG-coated GNR suspensions were successfully synthesized and characterized by different microscopy and analytical instrumentation techniques. The optical absorption spectra of CTAB-GNR (AR ~ 4) demonstrated two typical plasmon peaks, at 520 nm and 830 nm, respectively (Fig. 2A), while CTAB-GNR (AR ~ 2) showed the plasmon peaks at 520 nm and 640 nm, respectively (Fig. 2B).

**Figure 3:**
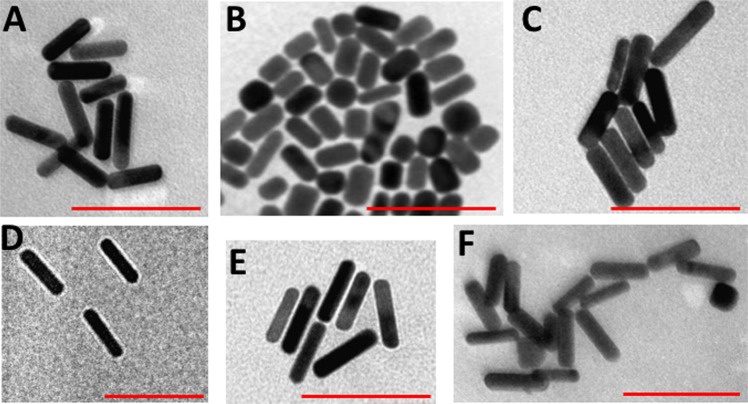
Transmission electron microscope images of GNR of different particle sizes and surface functionalities. (**A**) m-PEG-GNR (AR ~ 4), (**B**) m-PEG-GNR (AR ~ 2), (**C**) COOH-PEG-GNR (AR ~ 4), (**D**) DSPE-PEG-GNR (AR ~ 4), (**E**) Chol-PEG-GNR (AR ~ 4). (**F**) NH_2_-PEG-GNR (AR ~ 4). Scale: 100 nm.

Upon surface modifications with PEG-SH of different functionalities, no tailing or broadening was observed in the optical spectra, indicating excellent stability of the synthesized colloids. However, a slight shift of the longitudinal peaks of the functionalized GNR was identified related to the refractive index of the coating moieties or the suspending solvents (in case of toluene) (Fig. 2A–D). The zeta potential of CTAB-GNR and the modified nanorods confirmed their successful functionalization with different PEG-SH containing ligands. CTAB-GNR of AR ~ 4 and ~2 demonstrated positive zeta potentials (+57 and +58 mV, respectively, Fig. 2E), whereas m-PEG-GNR of AR ~ 4 and ~2 showed neutral surface charges (+2.3 and +1.8 mV, respectively, Fig. 2E). The zeta potential values were high for both NH_2_-PEG-GNR and COOH-PEG-GNR (+29.0 and −21.0, respectively, Fig. 2E). DSPE-PEG-GNR and Chol-PEG-GNR suspended in water demonstrated relatively low zeta potential values (−10.0 and +9.3, respectively; Fig. 2E). The average hydrodynamic diameter of CTAB-GNR of AR ~4 using DLS was ~52.0 nm, and it became ~69.7 nm and ~62.8 nm upon functionalization with DSPE-PEG-SH and Chol-PEG-SH, respectively. These shifts in hydrodynamic diameters suggest the formation of phospholipid-PEG and Chol-PEG layers covalently linked to GNR surface *via* thiol moieties (Fig. 2F). Besides, the hydrodynamic diameters for other modified GNR of AR ~ 4 and ~2 demonstrated slight increase in particles size upon surface modifications (Fig. 2F). The nanoparticles’ shape and size and the absence of GNR aggregates upon functionalization were also verified by TEM. TEM imaging of PEGylated GNR of AR ~ 4 demonstrated average diameters of 47.1 nm and 12.7 nm for length and width, respectively and average AR of ~3.7 (Fig. 3A), while m-PEG-GNR of AR ~ 2 showed average diameters of 33.1 nm and 15.2 nm for length and width, respectively and average AR of ~2.1 (Fig. 3B). Further, TEM images of DSPE-PEG-GNR, Chol-PEG-GNR and other modified GNR of AR ~ 4 demonstrated well-dispersed rod-shaped nanoparticles having similar particle sizes without significant aggregation (Fig. 3C–F).

The displacement of CTAB bilayer by DSPE-PEG-SH or Chol-PEG-SH was monitored and confirmed by ^1^H-NMR. The ^1^H-NMR chemical shifts of DSPE-PEG-SH and its corresponding DSPE-PEG-GNR, and that of Chol-PEG-SH and its corresponding Chol-PEG-GNR are similar (the assigned intense PEG peak, hydrocarbon peak and other peaks, Figures S1A,B and S2A,B, Supplementary Information). However, some 1H-NMR peaks were slightly broadened or shifted upon conjugation with GNR, in which the thiolated ligand forms Au—S with gold atoms. Monitoring the conjugation of thiolated molecules to GNP by ^1^H-NMR was described previously in the literature, where the ^1^H-NMR spectra demonstrated complicated changes upon binding of thiol terminated ligands to the surface of GNP. This was explained by the vicinity of the molecules to the GNP surfaces^43,44^.

Understanding the interaction of DSPE-PEG-SH or Chol-PEG-SH with CTAB-GNR is provided by comparing the ^1^H-NMR spectra of the thiolated ligands and the modified GNR with the ^1^H-NMR spectrum of CTAB-GNR (Figures S1 and S2, Supplementary Information). While many of the ^1^H-NMR peaks of CTAB overlapped with that of DSPE or cholesterol spectra, the γ-methyl peak for CTAB attached to GNR (δ ~ 3.39 ppm) is clearly distinct^45^. Upon addition of DSPE-PEG-SH or Chol-PEG-SH, a dramatic reduction of the intense γ-methyl peak of the CTAB-coated GNR at ~3.39 ppm, and a complete elimination of the small peak related to the protons attached to C1 of CTAB were observed in the spectra of DSPE-PEG-GNR and Chol-PEG-GNR. Also, a new sharp proton peak represents the repeated units of PEG at ~3.68 ppm appeared. An extra small peak at ~1.7 ppm of the ^1^H-NMR spectrum of CTAB-GNR is most likely due to a trace impurity^46^.

It is worth mentioning that CTAB-GNR suspension was centrifuged twice before addition of the thiolated ligands, and one more time after an overnight mixing with the thiolated ligands in order to remove free CTAB from the suspensions. Furthermore, the ligands were added to CTAB-GNR in excess to facilitate the CTAB displacement process. The above ^1^H-NMR results clearly demonstrate the successful displacement of nanorods-bound CTAB by the addition of DSPE-PEG-SH or Chol-PEG-SH. Similar results were demonstrated by Orendorff *et al*. using ^1^H-NMR to follow the interaction of a phospholipid with the CTAB-GNR composite^45^.

### Colloidal stability of GNR coated with PEG-SH-containing different functionalities upon human skin contact

**Figure 4:**
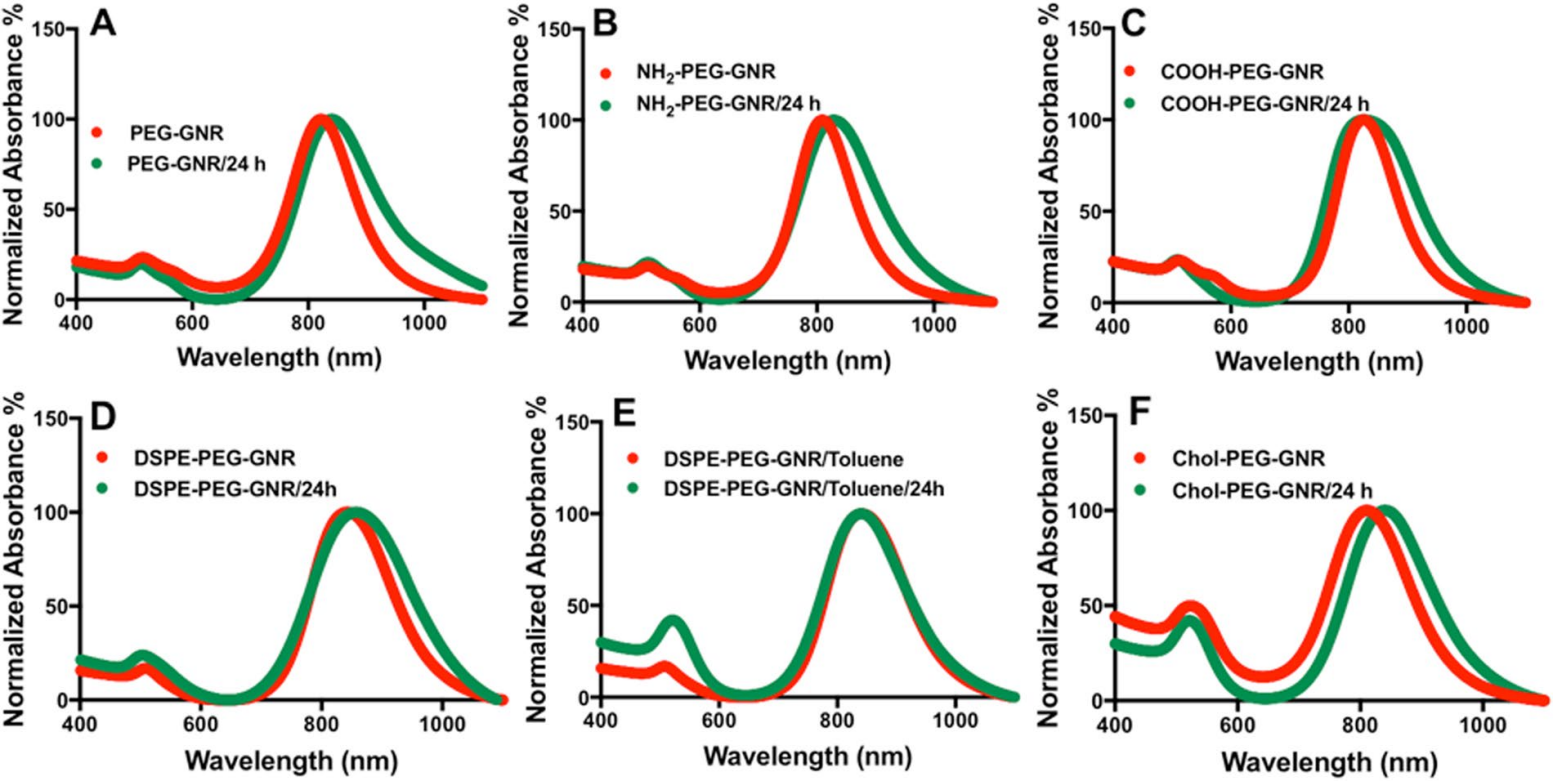
Optical absorption spectra of GNR of different surface functionalities (AR ~ 4) before (red lines) and after 24 hr of skin contact (green lines). (**A**) m-PEG-GNR, (**B**) NH_2_-PEG-GNR, (**C**) COOH-PEG-GNR, (**D**) DSPE-PEG-GNR. (**E**) Chol-PEG-GNR. All spectra reveal no significant tailing or broadening of the plasmon peaks that indicates excellent colloidal stability of GNR suspensions upon skin exposure.

The colloidal stability of many nanoparticles when interacting with skin is ignored in the majority of skin permeation and diffusion studies, which might be responsible for the observed conflicting results. Accordingly, prior to investigating the penetration of GNR into skin layers, their colloidal stability upon exposure to skin surface was examined. Previously, we have reported an enhanced aggregation of functionalized GNR with electrostatic-attracted cationic groups relative to their anionic PEGylated counterparts due to protein adsorption upon contact with human skin^42^. Herein, the colloidal stability of the surface modified GNR was evaluated upon exposure to human skin by inspecting their colloidal color changes, and by measuring their optical absorption spectra at zero time and after 24 hr of skin contact. Interestingly, all GNR of different functionalities containing PEG-SH demonstrated no color change and exhibited typical plasmon peaks with slight broadening of the longitudinal peaks after 24 hr of skin exposure (Fig. 4A–F). The colloidal stability of GNR suspensions was inspected also by recording the nanoparticles’ hydrodynamic size and zeta potential before and after skin exposure (Table 1). No significant differences in the particle sizes or zeta potentials were reported after 24 hr, which confirm the excellent colloidal stability of the nanorods upon human skin exposure.

**Table 1:** Hydrodynamic sizes measured by DLS, and effective surface charges of GNR of different surface functionalities before and after human skin contact for 24 hr. Data are given as mean ± SD (n = 3).

GNR	Hydrodynamic size (nm)		Surface charge (mV)	
	*Before skin contact*	*After skin contact*	*Before skin contact*	*After skin contact*
m-PEG-GNR	51.2 ± 2.1	53.5 ± 2.6	+2.3 ± 0.9	+0.9 ± 0.09
NH_2_-PEG-GNR	50.2 ± 3.2	54.1 ± 3.3	+30.0 ± 1.0	+20.3 ± 0.8
COOH-PEG-GNR	52.4 ± 0.9	55.1 ± 2.5	−22.2 ± 0.5	−26.8 ± 1.1
DSPE-PEG-GNR	67.8 ± 1.9	74.9 ± 1.7	−13.6 ± 0.7	−11.5 ± 0.8
Chol-PEG-GNR	65.6 ± 2.1	69.2 ± 2.1	+11.2 ± 2.3	+9.3 ± 2.6

The stabilization effect of PEG on GNR is well-demonstrated in the literature, where the presence of PEG layer stabilizes the nanoparticles and retards the adsorption of proteins onto their surfaces by steric repulsion^47^. Further, the presence of PEG-SH, especially for DSPE and cholesterol moieties, enhances their water disperse-ability^48^. It is important to mention that DSPE-PEG-SH and Chol-PEG-SH were added to GNR suspensions in excess, in order to provide a full coverage of GNR with thiolated ligands and to prevent their aggregation.

### The penetration and accumulation of GNR of different particle sizes and different surface functionalities into human skin layers

In order to preserve human skin structural integrity and to minimize artifacts and co-factors, the experimental human skin was used within a very short time of excision (5 days).

The interaction of CTAB-GNR with skin was not investigated in this study owing to their well-known significant cytotoxicity and poor colloidal stability^49^.

**Figure 5:**
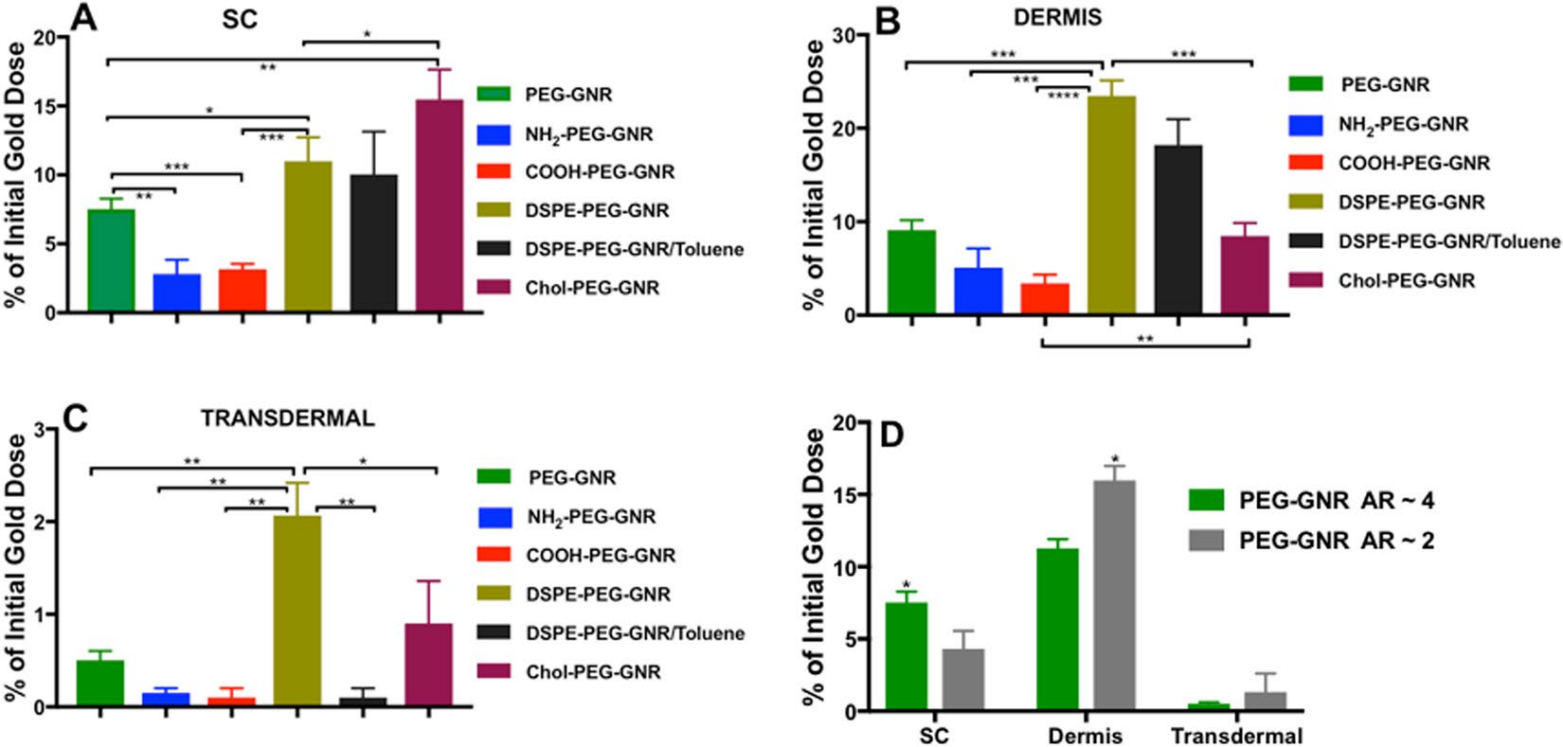
Percentages of GNR of different particle sizes and surface functionalities that accumulated into skin layers after 24 hr of incubation. (**A**) Percentages of GNR (AR ~ 4) accumulated into SC. (**B**) Percentages of GNR (AR ~ 4) accumulated into dermis. (**C**) Percentages of GNR (AR ~ 4) delivered transdermally into the acceptor media. (**D**) Percentages of PEG-GNR (AR ~ 4 *vs*. AR ~ 2) that accumulated into SC, dermis or delivered transdermally into the acceptor media. Data are presented as mean ± SD (n = 4). Unpaired t-test was used to assess the differences; *p < 0.05, **p < 0.01, ***p < 0.001 and ****p < 0.0001. AR: aspect ratio.

Human skin is composed of SC (made of proteins and lipids), epidermis and a more hydrophilic lower layer called dermis^50^. The preferential accumulation of GNR having the same particle size and different surface functionalities and zeta potential values into skin layers was evaluated quantitatively by measuring the amount of gold deposited into skin SC or dermis. Figure 5 illustrates the percentage of the initial gold dose of GNR that accumulated in SC and dermis and that delivered trans-dermally into the acceptor media. Chol-PEG-GNR were significantly accumulated into the SC (∼15.5%), compared to DSPE-PEG-GNR (∼8.0%), m-PEG-GNR (∼7.0%) or charged-GNR (NH_2_-PEG-GNR or COOH-PEG-GNR) (<5%)(Fig. 5A). Interestingly, DSPE-PEG-GNR revealed high percentage of accumulation into skin dermis (∼23.5%) compared to Chol-PEG-GNR (∼8.5%) or neutral charged m-PEG-GNR (∼11.0%) (Fig. 5B). However, charged-GNR with high zeta potential values (NH_2_-PEG-GNR and COOH-PEG-GNR) demonstrated minimal accumulation percentages into skin dermis (Fig. 5B). For GNR that were delivered trans-dermally into the acceptor media, DSPE-PEG-GNR present the highest percentage of transdermal delivery (∼2.0%), compared to other modified-GNR (Fig. 5C). PEGylated nanorods with a spheroid shape (AR of ~2) showed a significant accumulation into skin dermis compared to their counterparts having AR of ~4, while their accumulation into the SC was significantly less than that of nanorods with AR ~ 4. Both sizes of PEGylated nanorods revealed low concentrations in the acceptor media (Fig. 5D).

In order to probe the effect of the solvent on the penetration of GNR into skin, DSPE-PEG-GNR were suspended in water and in toluene. Although the penetration of DSPE-PEG-GNR-suspended in water into SC or dermis was slightly higher than that of DSPE-PEG-GNR-suspended in toluene, the difference was not statistically significant (Fig. 5A and B). In tune with other study, toluene does not have substantial enhancement effect on skin penetration, because toluene cannot extract ceramides, which are the main lipids constituents of SC^51^.

**Figure 6:**
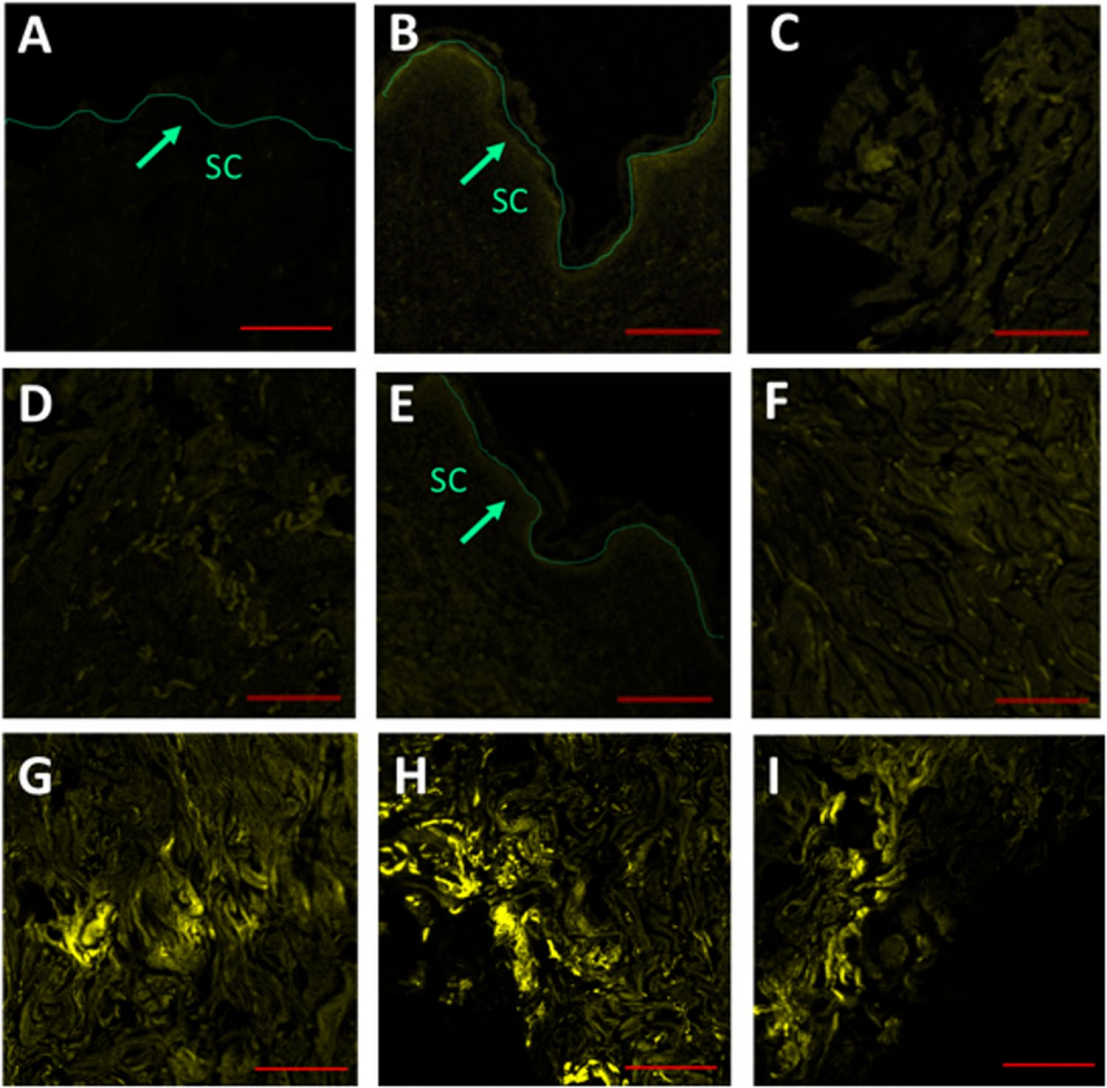
Confocal laser scanning microscopy images of skin samples pre-treated with DSPE-PEG-GNR or Chol-PEG-GNR. (**A**) SC of skin sample pre-treated with milli-Q water as a control. (**B**) SC of skin sample pre-treated with Chol-PEG-GNR. (**C**) High magnification of SC of skin sample pre-treated with Chol-PEG-GNR. (**D**) Dermis layer of skin sample pre-treated with Chol-PEG-GNR. (**E**) SC of skin sample pre-treated with DSPE-PEG-GNR. (**F**) High magnification of SC of skin sample pre-treated with DSPE-PEG-GNR. (**G**–**I**) Dermis layers of skin samples pre-treated with DSPE-PEG-GNR. Scales: **A**, **B** and **E** = 200 μm, **C**, **D**, **F**–**I** = 100 μm.

Furthermore, the preferential accumulation of GNR into skin layers was evaluated by confocal laser scanning microscopy, due to the unique light scattering properties obtained by GNP^52^. The control skin sample showed a negligible fluorescence background (Fig. 6A), while an appreciable fluorescence was perceived in the SC layers of skin incubated with Chol-PEG-GNR compared to DSPE-PEG-GNR (Fig. 6B,C,E,F). On contrast, enormous fluorescence was remarked in the dermis layer of skin treated with DSPE-PEG-GNR relative to Chol-PEG-GNR, which confirms the preferential accumulation of DSPE-PEG-GNR into skin dermis (Fig. 6G–I and D).

Figure S3, Supplementary Information, demonstrates the fluorescence of skin treated with m-PEG-GNR, COOH-PEG-GNR and NH_2_-PEG-GNR. Moderate fluorescence intensities were noticed in SC and dermis of skin treated with m-PEG-GNR (Figure S3A,B, Supplementary Information). On the other hand, low fluorescence intensities were recorded for the upper layers of skin pre-treated with charged-PEGylated GNR (COOH-PEG-GNR and NH_2_-PEG-GNR), which confirm the key role of the nanoparticles’ surface chemistry in determining their preferential accumulation into different skin layers (Figure S3C–F, Supplementary Information).

The fluorescence intensity of the confocal fluorescence images was quantified and graphed in order to correlate their results with ICP-OES findings. The estimated fluorescence intensities of skin treated with GNR of different surface functionalities are in agreement with their quantities measured by ICP-OES; where Chol-PEG-GNR and DSPE-PEG-GNR showed the highest accumulation percentages in SC and dermis, respectively (Figure 4SA,B, Supplementary Information).

**Figure 7:**
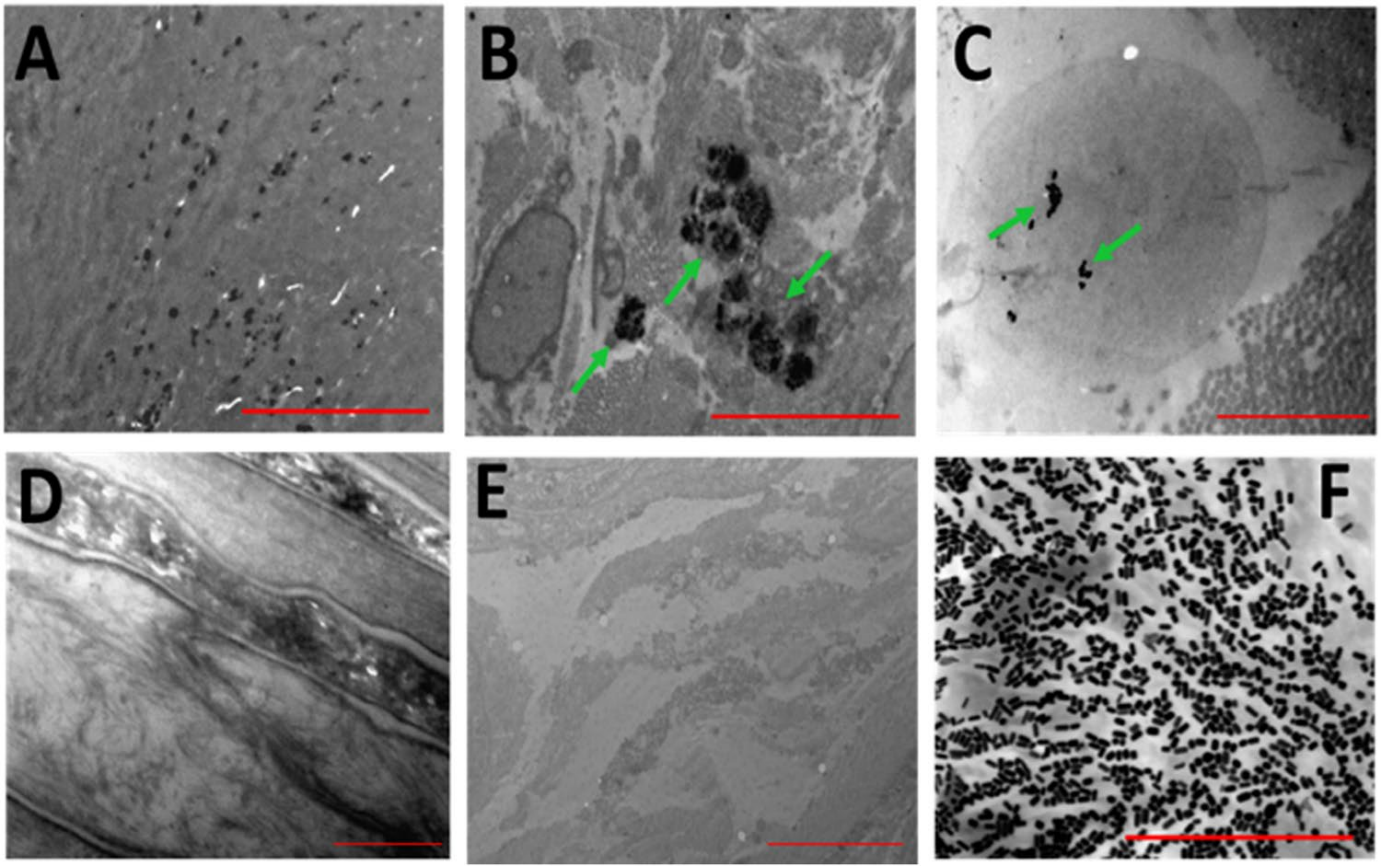
Transmission electron microscope images presenting the distribution of DSPE-PEG-GNR into skin layers. (**A**) SC showing the rod-shape of the penetrated GNR. (**B**,**C**) Clusters of GNR at skin dermis. (**D**,**E**) SC and dermis of skin treated with milli-Q water, respectively (as control). (**F**) Transmission electron microscope of GNR suspension to compare their shape and particle size to those accumulated into skin. Scales: **A**, **C**, **D** and F = 2 μm; (**B**,**E**) = 5 μm.

Moreover, the accumulation of GNR into the skin layers was visualized by TEM. DSPE-PEG-GNR are presented as black rod-shaped nanoparticles in the upper skin layers (Fig. 7A). In agreement with the previous results of ICP-OES and confocal fluorescence imaging, DSPE-PEG-GNR were highly deposited into the deep skin layers as small “aggregates” (Fig. 7B and C). The SC and dermis of control skin samples treated with milli-Q water demonstrated no characteristic dark rod-shaped nanoparticles (Fig. 7D and E, respectively). Figure 7F demonstrates a TEM image of GNR suspension that showed similarity between the particles’ shape and size to those accumulated into skin.

Understanding and evaluating the penetration of topically applied nanomaterials into skin layers have been the focus of many recent studies. For examples, zinc oxide nanoparticles accumulated in the SC, although they demonstrated *in vitro* toxicity^53,54^. Liquid colloidal systems such as nanoemulsions are enhancing the dermal drug delivery by disruption of SC lipid, however they have poor stability and could cause skin irritation^55,56^. Furthermore, solid lipid nanoparticles demonstrated a shape deformation after application to skin and enhancing the dermal penetration of the loaded drugs^57^. However, their low drug loading capacity and poor stability limit their applications^58^. The penetration and preferential accumulation of the nanomaterials into skin layers were strongly correlated to their sizes and surface functionalities. For examples, Prow *et al*. found that PEGylated quantum dots were deposited into the epidermis of skin and not dermis compared to PEG-NH_2_ or PEG-COOH which demonstrated no skin penetration at all^59^. Also, Zhang *et al*. have found that nanoparticles made of poly (d,l-lactic-co-glycolic acid) accumulated in the epidermis rather than the dermis, and their skin permeation was highly correlated to the particle size^60^.

The effect of phospholipids on the uptake of nanoparticles into cells or tissues was evaluated in the literature using liposomes as a model. Liposomes can enhance the transdermal drug delivery, although their penetration through SC is limited^61^. Liposomes have some disadvantages such as instability, low solubility, rapid elimination from the blood and leakage of the loaded drugs^61^. Compared to many other nano-systems, GNP are superior in synthesis and chemical stability, and their surfaces can be easily modified with different ligands at a density of 100-fold higher than that of liposomes^62^. In addition, GNP can be easily quantified and tracked within the biological systems owing to their high electron density^13,14^. Moreover, their biomedical applications extend beyond drug delivery vehicle to diagnosis and sensing applications, in addition to photothermal therapy.

Generally, the nano-bio interface is highly impacted by chemical and physical characteristics of the nano-system in addition to the properties of the biological barrier. In previous studies, it was demonstrated that phospholipids can penetrate and disturb the structure of SC lipids due to lipid mixing effect between phospholipids and SC lipids using liposomes as a model^63,64^. Kirjavainen *et al*., concluded that phospholipids acted as enhancers and increased the distribution of drugs into the SC by increasing the fluidity of SC lipids^65^. Based on the current results, we suppose that cholesterol and phospholipid enhanced the penetration of GNR through the upper skin layers due to their proposed modulation effect on the lipid contents of SC which consists mainly of ceramides, fatty acids, cholesterol and cholesteryl esters^3,4,7^. Also, we propose that the hydrophilic nature of the dermis probably retarded any further penetration of Chol-PEG-GNR into the deep skin layers compared to DSPE-PEG-GNR. These results agreed with our previous findings, where the penetration of hydrophobic polystyrene-thiol coated-GNR into skin dermis was limited by the hydrophilic nature of the dermis^34^. The preferential drastic accumulation of DSPE-PEG-GNR into deep skin layers was probably owing to their amphiphilic properties, which enhanced their distribution into the deep hydrophilic skin layers through SC. Similarly, Iannuccelli *et al*., have found that coating silica nanoparticles with lipids has drastically increased their penetration into human SC compared to the hydrophilic counterparts^66^. In addition to the drastic impact of phospholipids in cellular/tissues uptake, the colloidal stability was enhanced for many nanoparticles modified with a phospholipid bilayer^27,67^.

Neutral charged PEGylated GNR were distributed into upper and lower skin layers which is most likely due to their hydrophilic-hydrophobic properties and their penetration enhancing activity^68^. However, the charged PEGylated nanorods with high zeta potential values demonstrated the lowest percentage of accumulation into skin layers. These results are in concordance with previous studies using charged non-PEGylated nanoparticles^34,66^.

The accumulation potential of m-PEG-GNR of different aspect ratios was evaluated in this study to understand the impact of particle size on the interaction of GNR with human skin layers. Interestingly, PEGylated nanorods with AR of ~2 (spheroids) demonstrated high accumulation percentage into skin dermis compared to the PEGylated nanorods with AR of ~4. Several studies reported that as the gold nanospheres’ size decreases, their accumulation into skin are enhanced^17,69^. However, reports evaluating interaction of different sizes of GNR with skin are lacking in the literature. Fernandes *et al*. have found that the total percentage of GNR in skin was higher than that of gold nanospheres, however, no data regarding the percentage in each skin layer was provided^18^.

The results of the transdermal delivery of GNR into the acceptor media of Franz diffusion cells (FDCs) revealed that DSPE-PEG-GNR could be successfully employed for transdermal drug delivery *via* skin, although the percentage of GNR delivered transdermally was very low (∼2.0%).

The overall results demonstrated that preferential accumulation of GNR into human skin layers was strongly associated with their surface functionality.

### Antibacterial and Photothermal-based antibacterial activities of DSPE-PEG-GNR and Chol-PEG-GNR against *S. aureus*

The photothermal therapy of GNR is a cornerstone in their nanomedicine and biomedical applications^13^. As DSPE-PEG-GNR and Chol-PEG-GNR have demonstrated the highest percentages of accumulation into skin, we have investigated the antibacterial activity and photothermal-based antibacterial ablation effect of DSPE-PEG-GNR and Chol-PEG-GNR against *S. aureus*, the leading cause of soft tissues and skin infections^70^. The antibacterial effect of GNR was explored by estimating the minimum inhibitory concentrations (MIC) and percentage reduction of *S. aureus* viable count at the MIC values^71,72^. MIC is the lowest concentration of the agent that prevents the visible growth of a pathogen^71^.

Table 2 represents the MIC values of DSPE-PEG-GNR and Chol-PEG-GNR against *S. aureus*. Interestingly, phospholipid-coated GNR resulted in 4-log reduction (99.99%) in bacterial viable count at its MIC value (0.011 nM). However, cholesterol-coated GNR showed only 2-log reduction (99%) in bacterial viable count at its MIC value (0.75 nM). The coating materials (DSPE-PEG-SH and Chol-PEG-SH) demonstrated no antibacterial activity against *S. aureus*.

**Table 2:** Minimum inhibitory concentration (MIC) values of DSPE-PEG-GNR and Chol-PEG-GNR against *S. aureus* and the percentage and log reduction of *S. aureus* viable count at the MIC values. Data are presented as mean ± SD (n = 3). *SD for MIC values are zero.

GNR	MIC (nM)*	Reduction of viable count (%)	Log reduction
DSPE-PEG-GNR	0.011	99.99 ± 0.0025	4.0
Chol-PEG-GNR	0.75	99.56 ± 0.05	2.3

This is the first study to report the antibacterial activity of lipid-coated GNR against Gram positive bacteria such as *S. aureus*. Previously, phospholipid stabilized silver nanoparticles demonstrated a weak bactericidal activity against *S. aureus* (70%)^73^.

**Figure 8:**
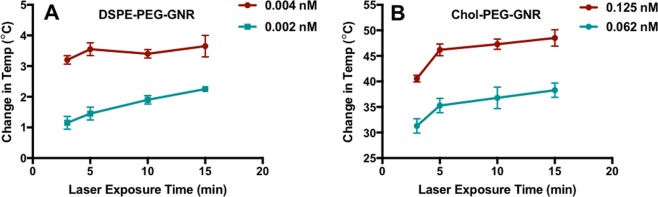
Temperature changes induced by NIR laser excitation of GNR suspensions over 15 min. (**A**) Temperature change of DSPE-PEG-GNR suspension at sub-MIC concentrations upon laser exposure. (**B**) Temperature change of Chol-PEG-GNR suspension at sub-MIC concentrations upon laser exposure. The initial temperature was 19.7 ± 0.4 °C. Data are presented as mean ± SD (n = 3).

Furthermore, the photothermal ablation effect of phospholipid and cholesterol-modified GNR against *S. aureus* was investigated in this study upon laser beam treatment over concentrations below the MIC values. The laser excitation time was set to 15 min since aggregation of the nanorods was observed after more than 15 min of laser exposure^35^. The changes in GNR suspension temperature upon exposure to the laser beam was recorded over time and the photothermal-based antibacterial activity was evaluated using the standard plate viable count method^72^. For DSPE-PEG-GNR, laser excitation resulted in no additional reduction in *S. aureus* viable count over concentrations below the MIC value (0.004 and 0.002 nM) compared to the combined effect of the controls (GNR or laser alone) (Table 3). A slight increase in DSPE-PEG-GNR suspensions’ temperature upon excitation with the laser was reported over 15 min relative to the initial temperature (Fig. 8A).

**Table 3:** Percentage and log reduction of *S. aureus* viable count after DSPE-PEG-GNR and Chol-PEG-GNR treatments with and without laser at sub-MIC concentrations. Data are presented as mean ± SD (n = 3).

	Reduction of viable count (%)	Log reduction
**DSPE-PEG-GNR**		
0.004 nM	91.0 ± 0.20	1.0
0.004 nM + Laser	98.0 ± 0.07	1.7
0.002 nM	93.8 ± 0.70	1.2
0.002 nM + Laser	99.0 ± 0.70	2.0
Laser	93.5 ± 2.2	1.2
**Chol-PEG-GNR**		
0.125 nM	93.0 ± 4.2	1.1
0.125 nM + Laser	99.99 ± 0.003	4.0
0.06 nM	86.5 ± 2.1	0.87
0.06 nM + Laser	99.6 ± 0.14	2.4
Laser	93.5 ± 2.2	1.2

On the other hand, Chol-PEG-GNR demonstrated an extra 2-log reduction in *S. aureus* viable count at concentration of 0.125 nM (sub-MIC) compared to the combined effect of the controls (GNR or laser alone) (Table 3). A significant increase in Chol-PEG-GNR suspension’s temperature over 15 min of laser exposure was recorded relative to the initial temperature (~65 *vs*. ~19.7 °C) (Fig. 8B). Chol-PEG-GNR at 0.06 nM (sub-MIC) did not result in any extra bactericidal activity compared to the combined effect of the controls although the temperature of the GNR suspension was increased by 35 °C over 15 min of laser exposure compared to the initial temperature (~46 *vs*. ~19.7 °C) (Fig. 8B).

The recorded localized heat obtained upon NIR excitation correlates well with the percentage of *S. aureus* viable count reduction which confirms the role of photothermal therapy in bacterial cells ablation. However, the photothermal antibacterial activity was not significant at the sub-MIC values of DSPE-PEG-GNR suspension, due to the low concentration of the photothermal-triggering species, the DSPE-PEG-GNR at these concentrations. The photothermal antibacterial activity of Chol-PEG-GNR at 0.06 nM resulted in no additional reduction of *S. aureus* viable count although the generated localized heat was increased by 35 °C at this concentration. This emphasizes the key role the surface functionality of GNR plays in delivering the nanorods into bacteria and consequently maximizing the photothermal effect. For effective photothermal therapy, the nanorods should be at least adsorbed onto the targeted cell surface, so the generated heat obtained upon NIR laser excitation can cause the required cell damage^15^. Accordingly, we suppose that the phospholipid utilized to modify the surface of GNR has enhanced the adsorption of the nanorods into the bacterial membrane and might facilitated their bacterial uptake and subsequently their bactericidal activity. That is why DSPE-PEG-GNR demonstrated very low MIC value compared to Chol-PEG-GNR (0.011 *vs*. 0.75 respectively; ~68 folds).

The antibacterial and photothermal-based antibacterial activities of m-PEG-GNR were investigated in our previous work. PEGylated GNR have MIC value of ~0.45 nM against *S. aureus* and demonstrated 4-log reduction in *S. aureus* viable count at concentration range of 0.06–0.25 nM upon treatment with NIR laser beam light^35,37^.

The photo-thermolysis of bacteria employing GNR was described in the literature, however, the photothermal ablation activity of lipid-coated nanorods was not discussed before. Millenbaugh *et al*. reported that the laser-activated gold nanospheres conjugated with antibodies reduced *S. aureus* viability to 31%^74^, which is a very weak effect in comparison to what is reported in the current study.

### Imaging of *S. aureus* pre-treated with Chol-PEG-GNR or DSPE-PEG-GNR by TEM

**Figure 9:**
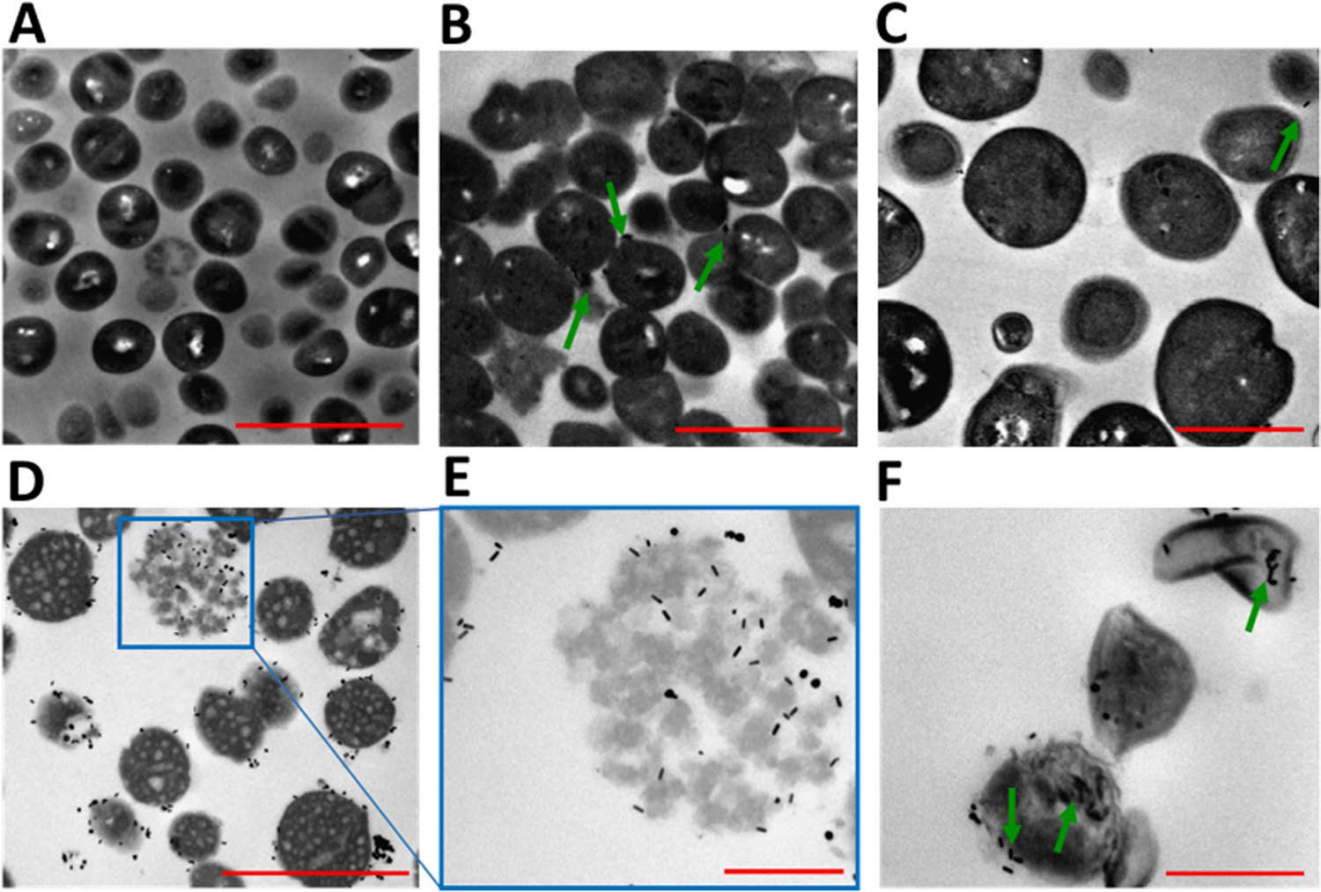
Transmission electron microscope images of *S. aureus* pre-treated with GNR. (**A**) Untreated *S. aureus* as control. (**B**,**C**) *S. aureus* treated with Chol-PEG-GNR. (**D**–**F**) *S. aureus* treated with DSPE-PEG-GNR. Arrows indicate the presence of GNR. Scales: (**A**,**B** and **D**): 2 μm, (**C**): 1 μm and (**E**,**F**): 0.5 μm.

The interaction of Chol-PEG-GNR or DSPE-PEG-GNR with *S. aureus* was further investigated using TEM imaging. Figure 9A represents a TEM image of untreated *S. aureus* as a control and indicates the intact shape of bacterial cells. Chol-PEG-GNR did not result in significant damage to *S. aureus* as presented in Fig. 9B,C. However, *S. aureus* pre-treated with DSPE-PEG-GNR demonstrated significant decrease in number and revealed notable bacterial uptake of the nanorods as presented in Fig. 9D–F. In addition, *S. aureus* showed significant disintegration and lysis upon treatment with DSPE-PEG-GNR as presented in Fig. 9D–F. The TEM images correlate well with the antibacterial activity of GNR, and clearly justified the potent bactericidal effect of DSPE-PEG-GNR, where phospholipid drastically enhanced the bacterial uptake and toxicity of GNR.

In conclusion, the preferential accumulation of GNR into skin layers was strongly associated with their surface chemical decoration. Phospholipids drastically enhanced GNR accumulation in skin dermis *via* SC, while cholesterol coated GNR were confined to the upper layers of SC with a minimum accumulation in the lower hydrophilic skin dermis. DSPE-PEG-GNR and Chol-PEG-GNR demonstrated reasonable antibacterial and photothermal-based bactericidal activities against *S. aureus*, the most common skin pathogen. Based on these findings, DSPE-PEG-GNR or Chol-PEG-GNR could be considered as a promising nano-therapy for skin diseases such as acne and skin infections.

## Materials and Methods

### Materials and instrumentations

Ascorbic acid (99%) chloroauric acid (HAuCl_4_.3H_2_O, 99.9%), methoxy-polyethylene glycol-thiol (m-PEG-SH, MW ~2000 g/mole), silver nitrate (AgNO_3_, 99%), carboxylic acid PEG-thiol (COOH-PEG-SH, MW ~2000 g/mole), sodium borohydride (NaBH_4_, 99%), amine PEG-thiol (NH_2_-PEG-SH, MW ~2000 g/mole), cetyltrimethylammonium bromide (CTAB, 99%), gold standard for ICP (1000 PPM) and glutaraldehyde solution were obtained from Sigma-Aldrich Chemicals, USA. 1,2-distearoyl-sn-glycero-3-phosphoethanolamine-N-PEG-SH (DSPE-PEG-SH, MW~2000 g/mole) and cholesterol-PEG-SH (MW~2000 g/mole) were obtained from Nanosoft Polymers, USA. 96 well-plates were purchased from Greiner bio- one, Germany. Nutrient agar, Mueller–Hinton broth, *S. aureus* ATCC 29213 were obtained from Oxoid, UK.

The following instruments were used in the study: Spectrophotometer (UV-1800, Shimadzu, Japan) was used for measuring the optical absorption spectra of GNR. Nicomp Nano Z3000 zeta potential/particle size analyzer (CA, USA). Versa 3D, FEI, Holland was used for TEM imaging. Hettich EBA 12 Centrifuge (Gemini BV, Netherland) was used for GNR centrifugation. Confocal laser scanning microscopy, LSM 780 (Carl Ziess, Germany) was used for skin samples imaging. Optima 2000 DV (ICP-OES, PerkinElmer Corporation of USA) was used for gold analysis. All proton nuclear magnetic resonance (^1^H-NMR) spectra were measured at 19.15 °C in Deuterated chloroform (CDCl_3_) using Bruker AVANCE 300 MHz NMR instrumentation, Germany, operating at 300.13 MHz. Franz diffusion cells (FDCs), 1.0 cm² area were obtained from PermeGear Inc., USA. TEM grids (Formvar-coated) were obtained from Electron Microscopy Sciences, USA. Laser diode, Max power output ~9 W, 808 nm was purchased from OSTECH, Germany. A digital thermometer probe was purchased from FLIR Commercial Systems Inc., USA.

### Methods

#### Chemical synthesis of CTAB-coated GNR (CTAB-GNR) of aspect ratio (AR) ~4 and ~2

CTAB-GNR were prepared using CTAB as a surfactant as described previously^34^. Briefly, NaBH_4_ was added to a mixture of CTAB and gold solution to form seeds. For growth of GNR, AgNO_3_ (1.3 mL, 0.01 M) was added to a solution of CTAB to acquire GNR with AR of ~4. After that, HAuCl_4_ solution, ascorbic acid and the seeds were added to the growth solution and incubated at 27 °C overnight. GNR suspension was spun down twice by centrifugation at 12000 rpm for 15 min and re-suspended in milli-Q water. The previous procedure was repeated using 0.3 mL of AgNO_3_ to obtain CTAB-GNR of AR ~ 2.

#### Surface modification of GNR with m-PEG-SH (PEG-GNR)

A volume of 1.0 mL of aqueous solution of m-PEG-SH (15 mg/mL) was transferred to 10.0 mL of CTAB-coated GNR suspension and mixed overnight. The PEGylated GNR suspension was spun down twice by centrifugation at 10 000 rpm for 10 min and re-suspended in milli-Q water.

#### Surface modification of GNR with anionic COOH-PEG-SH (COOH-PEG-GNR)

A volume of 1.0 mL of aqueous solution of COOH-PEG-SH (15 mg/mL) was transferred to 10.0 mL of CTAB-coated GNR suspension and mixed overnight. The coated GNR suspension was spun down twice by centrifugation at 10 000 rpm for 10 min and re-suspended in milli-Q water.

#### Surface modification of GNR with cationic NH_2_-PEG-SH (NH_2_-PEG-GNR)

A volume of 1.0 mL of aqueous solution of NH_2_-PEG-SH (15 mg/mL) was transferred to 10.0 mL of CTAB-coated GNR suspension and mixed overnight. The coated GNR suspension was spun down twice by centrifugation at 10 000 rpm for 10 min and re-suspended in milli-Q water.

#### Surface modification of GNR with DSPE-PEG-SH suspended in water (DSPE-PEG-GNR/water)

A volume of 1.0 mL of aqueous solution of DSPE-PEG-SH (20 mg/mL) was transferred to 10.0 mL of CTAB-coated GNR suspension and mixed overnight. The coated GNR suspension was spun down twice by centrifugation at 10 000 rpm for 10 min and re-suspended in milli-Q water.

#### Surface modification of GNR with DSPE-PEG-SH suspended in toluene (DSPE-PEG-GNR/toluene)

DSPE-PEG-GNR suspended in toluene were prepared by ligand exchange method^34^. The thiolated DSPE-PEG polymer was transferred from water to dichloromethane (DCM) and the dried DSPE-PEG-GNR were suspended in toluene using sonication for 20 min.

#### Surface modification of GNR with Chol-PEG-SH (Chol-PEG-GNR)

A volume of 1.0 mL of aqueous solution of Chol-PEG-SH (20 mg/mL) was transferred to 10 mL of CTAB-coated GNR suspension and mixed overnight. The coated GNR suspension was spun down twice by centrifugation at 10 000 rpm for 10 min and suspended in milli-Q water.

#### ^1^H NMR analysis of CTAB-GNR, DSPE-PEG-GNR and Chol-PEG-GNR

Samples were dissolved in CDCl_3_ using a Shigemi NMR microtube. Sodium trimethylsilyl-[2,2,3,3-d4]-propionate (TSP) was employed as an internal standard. An acquisition time of 3.11 s, a relaxation delay of 6 s and 128 transients of a spectral width of 8333.3 Hz were recorded into 65 k time domain points.

#### Characterization of GNR coated with PEG-SH-containing different functionalities

The synthesized GNR suspensions were characterized by optical absorption spectroscopy, DLS, effective surface charge, TEM and ^1^H-NMR.

#### Preparation of human skin samples

Human skin samples were received from a healthy female aged 32 years after undergoing an abdominal plastic surgery. Informed consent was obtained from the donor for study participation and the experimental protocols were approved by Al-Zaytoonah University of Jordan Research Ethics Committee (ZUJ-RES 2018/64/04) in adherence to Helsinki Guidelines.

The obtained skin was cleaned from the subcutaneous fat and stored at −80 °C till use^34^.

#### The colloidal stability of GNR of different surface functionalities upon contact with human skin surface

A volume of 400 µL (4.0 nM) of the following GNR suspensions: m-PEG-GNR, COOH-PEG-GNR, NH_2_-PEG-GNR, DSPE-PEG-GNR, and Chol-PEG-GNR were placed into the donor chambers of FDCs. Normal saline (pH ∼7.4) was added to the receiver chambers and the FDCs were incubated at 37 °C for 24 hr.

Samples of the applied GNR were taken after 24 hr of skin exposure, and their colloidal stability was evaluated by inspecting their colloidal color, UV-vis absorption spectra, hydrodynamic size and effective surface charge.

#### Skin permeation study of GNR of different particle sizes and surface functionalities

A volume of 400 µL (4.0 nM) of the following GNR suspensions: m-PEG-GNR (AR ~ 4), m-PEG-GNR (AR ~ 2), COOH-PEG-GNR, NH_2_-PEG-GNR, DSPE-PEG-GNR/water, DSPE-PEG-GNR/toluene, and Chol-PEG-GNR were placed into the donor chambers of FDCs. Normal saline (pH ∼7.4) was added to the receiver chambers and the FDCs were incubated at 37 °C for 24 hr.

#### Determination of gold quantities accumulated into skin layers using ICP-OES

After 24 hr incubation, the treated skin samples described in the previous section were prepared for gold analysis by ICP-OES as described previously^34^. Briefly, for each skin sample, the treated skin surface was swabbed using medical cotton to remove the excess amount of GNR. After that, SC layers were collected and separated from the dermis layer by the tape stripping technique using 20 adhesive tapes. The collected skin layers were lysed with 2.0 mL aqua regia (1HNO_3_:3HCL), diluted with milli-Q water, filtered using 0.22 μm filters and their gold content was measured by ICE-OES.

The analysis conditions were optimized as described previously^34^. Gold ions were measured at 242.795 nm and the gold concentration (mg/L) was estimated using a standard calibration curve of gold standard for ICP (0.1–10.0 ppm). The results presented as mean ± SD from at least three independent experiments.

#### Imaging of skin samples pre-treated with GNR of different surface functionalities using confocal laser scanning microscopy

Cleaned human skin samples were incubated for 24 hr with 400 µL (4.0 nM) of DSPE-PEG-GNR, Chol-PEG-GNR, m-PEG-GNR, NH_2_-PEG-GNR, COOH-PEG-GNR or milli-Q water (as a control) using FDCs. The skin samples were processed and fixed as thin slices (5 μm) on slides for confocal fluorescence imaging as described previously^38^. The optical excitation and emission wavelengths were 514 and 532 nm, respectively and the image size was around 424.27 μm × 424.27 μm.

The obtained images were analyzed by ImageJ 1.52a software (National Institute of Health, USA), and the fluorescence intensities were estimated as an average fluorescence intensity of at least three images, graphed and provided as charts.

#### Imaging of skin samples pre-treated with DSPE-PEG-GNR and PEG-GNR by TEM

Skin samples treated with DSPE-PEG-GNR or milli-Q water (as a control) for 24 h were imaged using TEM to evaluate the penetration and accumulation pattern of GNR. The samples were fixed in glutaraldehyde solution (3%) and the fixed tissues were prepared for TEM imaging as described previously^38^. Briefly, tissues were post-fixed in osmium tetraoxide buffer solution and were dehydrated in ethanol solutions, loaded in epoxy resin, sectioned into ~70 nm thin sections, fixed onto the grids and visualized by TEM.

#### Determination of MIC values of DSPE-PEG-GNR and Chol-PEG-GNR

The MIC values of DSPE-PEG-GNR and Chol-PEG-GNR against *S. aureus* were determined using the two-fold broth microdilution method^71^. A volume of 150 μL of Mueller–Hinton broth was placed in the wells and the GNR suspension (150 μL, 4.0 nM) was added to the first well. Then, serial dilutions (double fold) were performed throughout the plate. A 15 μL of MacFarland adjusted overnight culture of *S. aureus* was added to each well to obtain an average inoculum size of *ca*. 1.0 × 10^6^ CFU/mL. The plates were incubated overnight at 37 °C, and the MIC value was calculated as the average concentration of the well having turbidity and that clear^71^. The previous MIC set up was used to determine the percentage and log reduction in *S. aureus* using the standard plate viable count method at the last well showing no bacterial growth^72^. The antibacterial activity of the coating materials (DSPE-PEG-SH and Chol-PEG-SH; 1.0 mg/mL) was performed using the method described in this section.

#### Photothermal-based antibacterial activity of DSPE-PEG-GNR and Chol-PEG-GNR

We followed a previous protocol in evaluating the photothermal-based antibacterial activity of DSPE-PEG-GNR and Chol-PEG-GNR against *S. aureus*^35^. Briefly, bacterial inoculated wells (*ca*. 1.0 × 10^6^ CFU/mL) containing DSPE-PEG-GNR at concentrations of 0.004 and 0.002 nM (sub-MIC values), Chol-PEG-GNR at concentrations of 0.125 and 0.06 nM (sub-MIC values) and the negative control (no GNR) were incubated at 37 °C for 10 min and then excited with a laser beam (808 nm, ~3 W/cm^2^), at an area of ~0.5 cm^2^ for 15 min. The same above set up plate was incubated in dark condition without laser for 25 min at 37 °C. The percentage and log reduction in *S. aureus* viable count at the sub-MIC values were estimated using the standard plate viable count method^72^.

#### Imaging of *S. aureus* pre-treated with DSPE-PEG-GNR and Chol-PEG-GNR by TEM

A mixture of an overnight cultured *S. aureus* (1.0 mL, 1.0 × 10^6^ CFU/mL) and DSPE-PEG-GNR or Chol-PEG-GNR (200 μL, 4.0 nM) was incubated for 4 hr. Then, the mixture was spun down and the bacterial pellets were fixed in 3% glutaraldehyde and processed as described previously^35^. Sections of 70 nm thickness were obtained and fixed onto Formvar copper grids and imaged by TEM. Untreated bacteria fixed as previously described was used as a control.

#### Statistical analysis

Unpaired t-test was employed for statistical analysis employing GraphPad Prism, version 7.0. When p < 0.05, the results are considered significant.

## Supplementary information

Supplementary Dataset

## Data Availability

The datasets generated and/or analyzed during the current study are available from the corresponding author on reasonable request.
