# Supplementary material for: Preferential Accumulation of Phospholipid-PEG and Cholesterol-PEG Decorated Gold Nanorods into Human Skin Layers and Their Photothermal-Based Antibacterial Activity
Source: Sci Rep. 2019 Apr 8;9:5796. doi: 10.1038/s41598-019-42047-7 (PMC6453979; doi:10.1038/s41598-019-42047-7)
Supplement: Supplementary file 1 — Supplementary Dataset [file 41598_2019_42047_MOESM1_ESM.pdf]

Supplementary Information

Preferential Accumulation of Phospholipid-PEG and Cholesterol-PEG Decorated Gold Nanorods into Human Skin Layers and their Photothermal-Based Antibacterial Activity

Nouf N. Mahmoud[1*], Ala A. Alhusban[1], Jamila Isabilla Ali[1], Amal G. Al-Bakri[2], Rania Hamed[1], Enam A. Khalil[2]

[1] Department of Pharmacy, Faculty of Pharmacy, Al-Zaytoonah University of Jordan, Amman 11733, Jordan.

[2] Department of Pharmaceutics & Pharmaceutical Technology, School of Pharmacy, The University of Jordan, Amman 11942, Jordan.

**Corresponding author*: E-mail: nouf.mahmoud@zuj.edu.jo

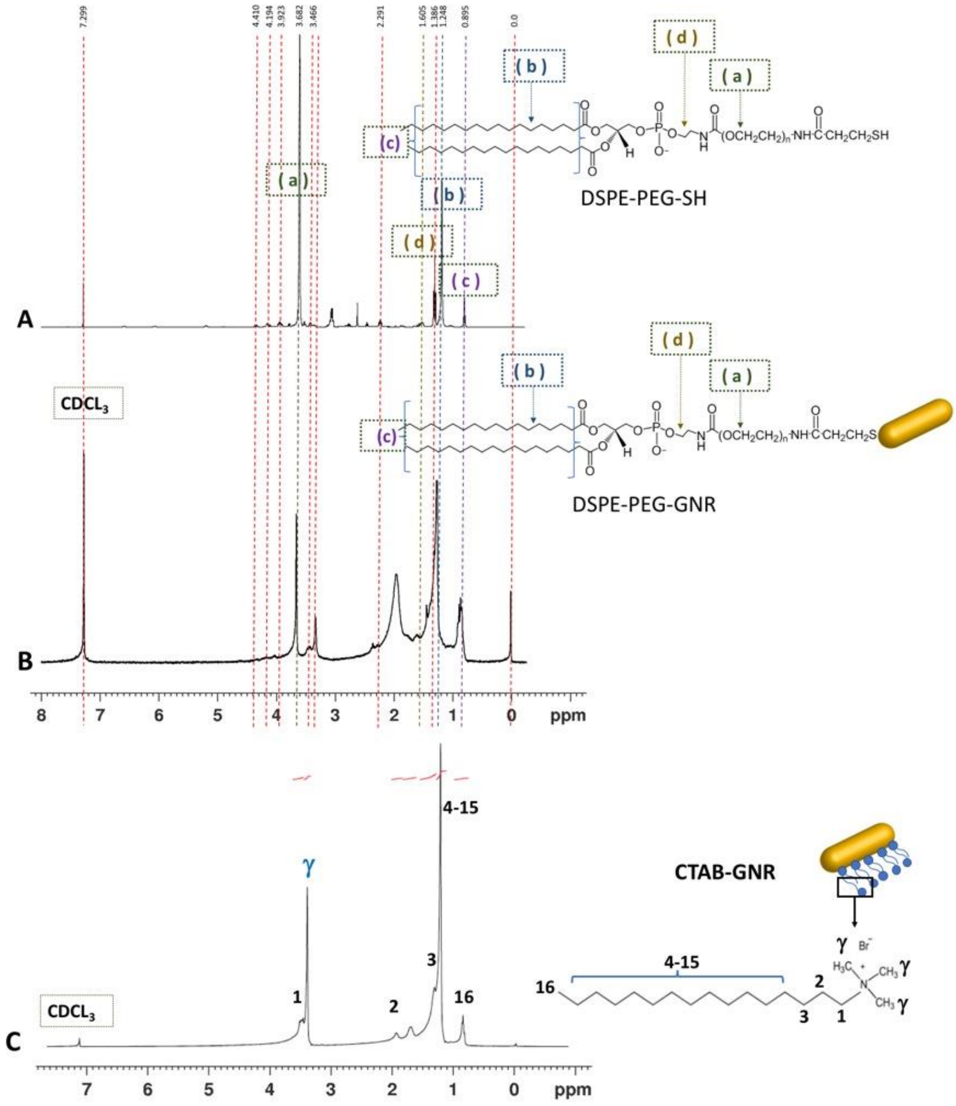

Figure S1. ^{1}H-NMR spectra of DSPE-PEG-SH, DSPE-PEG-GNR and CTAB-GNR. Combined ^{1}H-NMR spectra of **A.** DSPE-PEG-SH and **B.** DSPE-PEG-GNR show the signals similarity of characteristic chemical shift values with slightly broadened and shifted peaks upon attachment to GNR; (a) a characteristic sharp peak of repeated units of PEG at ~ 3.68 ppm; (b) a characteristic sharp peak of methylene groups of DSPE at ~ 1.25 ppm; (c) a characteristic peak of the methyl group of DSPE at ~ 0.89 ppm; (d) a characteristic peak of the methylene groups attached to amide at 1.60 ppm. **C.** ^{1}H-NMR spectrum of CTAB-GNR showed a

characteristic sharp peak of the γ-methyl protons at ~ 3.39 ppm that was reduced dramatically upon displacement of CTAB by DSPE-PEG-SH. All the spectra were acquired in $CDCL_3$.

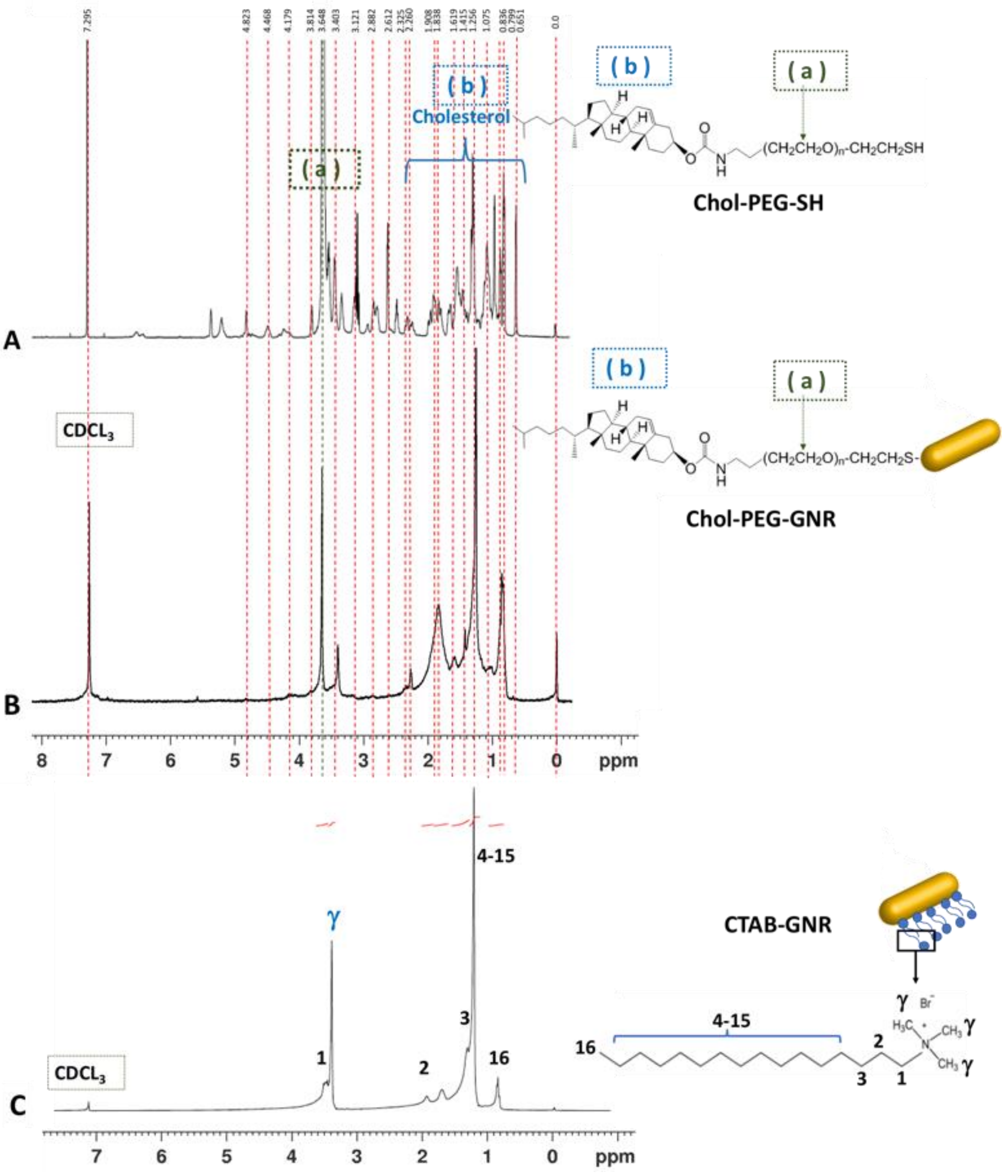

Figure S2. ^{1}H-NMR spectra of Chol-PEG-SH, Chol-PEG-GNR and CTAB-GNR. Combined ^{1}H-NMR spectra of **A.** Chol-PEG-SH and **B.** Chol-PEG-GNR show the signals similarity of characteristic chemical shift values with slightly broadened and shifted peaks upon attachment to GNR; (a) a characteristic sharp peak of repeated units of PEG at ~ 3.68 ppm; (b)

Characteristic peaks of cholesterol protons. **C.** ^{1}H-NMR spectrum of CTAB-GNR showed a characteristic sharp peak of γ-methyl protons at ~ 3.39 ppm that was reduced dramatically upon displacement of CTAB by Chol-PEG-SH. All the spectra were acquired in $CDCL_3$.

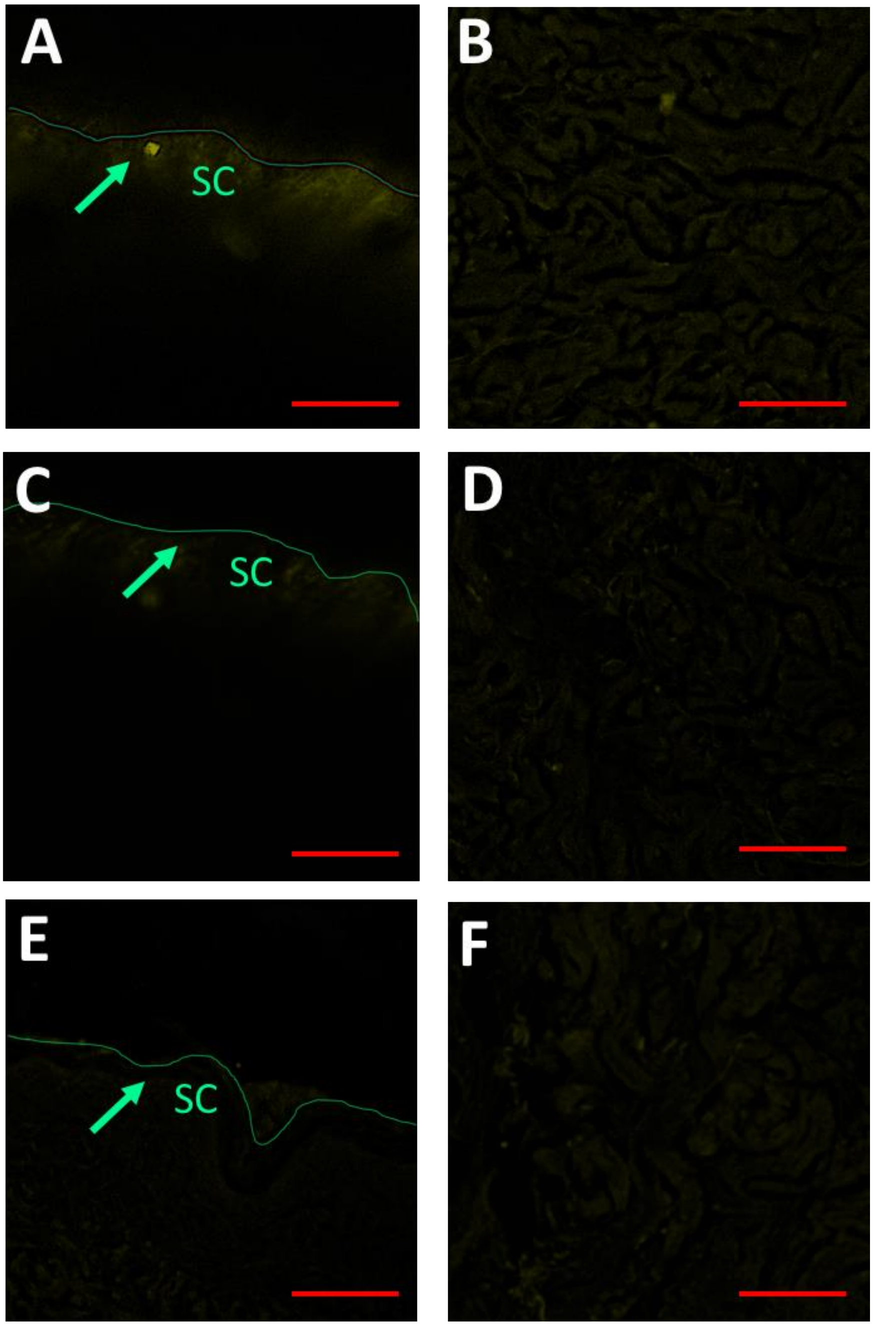

Figure S3. Confocal fluorescence microscopy images of skin samples pre-treated with m-PEG-GNR, NH_2-PEG-GNR and COOH-PEG-GNR. **A & B.** SC and dermis of skin pre-treated with m-PEG-GNR, respectively. **C & D.** SC and dermis of skin pre-treated with NH_2-PEG-GNR respectively. **E & F.** SC and dermis of skin pre-treated with COOH-PEG-GNR, respectively.

All the images showed low fluorescence intensity of the neutral or charged PEGylated GNR applied to skin samples. Scales = A, C and E = 200 μm, B, D and F = 100 μm.

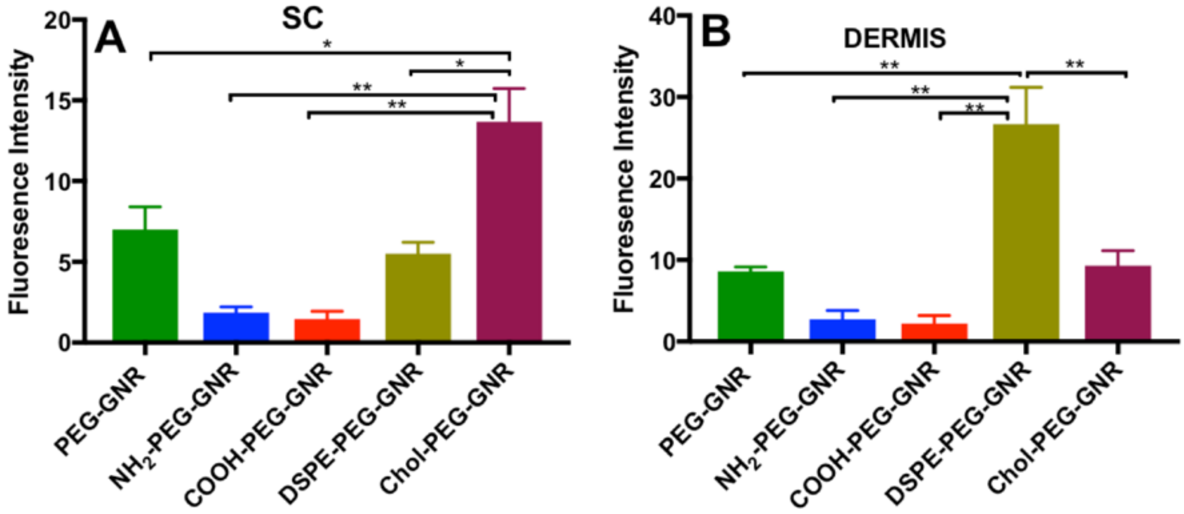

Figure S4. Fluorescence intensities of GNR of different surface functionalities that accumulated into: **A.** Stratum corneum layers, and **B.** Dermis layer. Data are given as mean ± SD (n = 3). Unpaired t-test was used to evaluate the differences; * $p < 0.05$, ** $p < 0.01$.
